# Editorial on the Special Issue “Geometry Reconstruction from Images (2nd Edition)”

**DOI:** 10.3390/jimaging11100339

**Published:** 2025-09-30

**Authors:** Daniel Meneveaux

**Affiliations:** XLIM Institute, UMR CNRS 7252, University of Poitiers, 86073 Poitiers, France; daniel.meneveaux@univ-poitiers.fr

In recent decades, research has produced impressive methods for recovering geometric information from real objects [1,2], laying the fundamental foundations for further studies in robotics, industry, medicine, architecture, and visualization approaches. The subject remains a very active field in computer vision, among many other scientific areas. Research advances have long been transferred to industry, but new trends and challenges continuously emerge [3,4], with new sensors, faster computation hardware, and the growing demand for increasingly accurate detail capture. Furthermore, applications for the general public are now appearing, for instance with 3D reconstruction available on mobile phones.

Alongside visualization techniques, machine learning has also increased the quality of reconstruction methods, with approaches such as NeRFs or Gaussian Splatting, which are addressed in this Special Issue. Some specific aspects still require more in-depth fundamental research, such as the management of specular surfaces or accuracy issues in underwater environments.

The 11 articles published in this second edition (https://www.mdpi.com/journal/jimaging/special_issues/55A1X64G0H, accessed on 25 September 2025) tackle several very interesting challenges: reconstructions of objects known for their complexity [5], underwater environments where distortions make depth estimation difficult, interactive systems and dynamic scenes, analyses of existing reconstruction techniques [6], and deep learning approaches.

List of contributions:

The first article, “Adaptive High-Precision 3D Reconstruction of Highly Reflective Mechanical Parts Based on Optimization of Exposure Time and Projection Intensity” by Ci He, Rong Lai, Jin Sun, Kazuhiro Izui, Zili Wang, Xiaojian Liu, and Shuyou Zhang, focuses on reconstructing mechanical parts with highly reflective surfaces. The proposed method relies on an adaptive 3D reconstruction approach that optimizes exposure time and projection intensity, while being further adjusted to the linear dynamic range of the hardware. The resulting image sequence is fused using a combination of a Genetic Algorithm and the Stochastic Adam optimizer to maximize image information entropy. The authors experimentally validate their approach on three sets of typical mechanical components, each with diverse geometric characteristics and varying levels of complexity.

The second article, “Impact of Data Capture Methods on 3D Reconstruction with Gaussian Splatting”, by Dimitar Rangelov, Sierd Waanders, Kars Waanders, Maurice van Keulen, and Radoslav Miltchev, investigates how different filming techniques influence the quality of 3D reconstructions for indoor crime scene investigations. The authors examine the impact of factors such as camera orientation, filming speed, data layering, and scanning path on the detail and clarity of 3D reconstructions using Neural Radiance Fields (NeRFs) and Gaussian Splatting. They identify optimal filming methods that help reduce noise and artifacts, and provide valuable guidelines for professionals in forensics, architecture, and cultural heritage preservation to capture realistic, high-quality 3D representations. The study also highlights opportunities for future research, particularly in exploring other algorithms, camera parameters, and real-time adjustment techniques.

In the third article, “Robot-Based Procedure for 3D Reconstruction of Abdominal Organs Using the Iterative Closest Point and Pose Graph Algorithms”, by Birthe Göbel, Jonas Huurdeman, Alexander Reiterer and Knut Möller, a procedure is proposed for a robot-based multi-view 3D reconstruction with pose optimization algorithms. In this work, a robotic arm and a stereo laparoscope build the experimental setup. The procedure includes stereo matching for depth measurement and the multiscale color iterative closest point algorithm, along with multiway registration for pose optimization. The procedure is evaluated quantitatively and qualitatively on ex vivo organs. The proposed procedure leads to a plausible 3D model, without hand–eye calibration.

The article “Fitting Geometric Shapes to Fuzzy Point Cloud Data”, by Vincent B. Verhoeven, Pasi Raumonen, and Markku Åkerblom, presents procedures and analysis on the reconstruction of geometry-derived data and its associated uncertainty. Instead of treating the data as a discrete point cloud, the authors consider it as a continuous fuzzy point cloud. They introduce a novel approach based on the expected Mahalanobis distance, which is illustrated using laser scanning data of a cylinder. Its performance is compared to that of the conventional least squares method, both with and without random sample consensus (RANSAC). The proposed method achieves a more accurate geometric fit, albeit generally with greater uncertainty, and demonstrates strong potential for geometry reconstruction from laser-scanned data.

The article “Arbitrary Optics for Gaussian Splatting Using Space Warping”, written by Jakob Nazarenus, Simin Kou, Fang-Lue Zhang, and Reinhard Koch, addresses the camera models employed in the context of 3D Gaussian Splatting. The authors propose a method to handle arbitrary camera optics, such as highly distorting fisheye lenses. Their approach applies a differentiable warping function to the Gaussian scene representation. They also introduce a learnable skybox for the specific case of outdoor scenes.

The article “A Mathematical Model for Wind Velocity Field Reconstruction and Visualization Taking into Account the Topography Influence”, by Guzel Khayretdinova and Christian Gout, proposes a global model for vector field approximation from a given finite set of vectors (corresponding to wind velocity fields or marine currents). The minimization process relies on a Hilbert space energy functional that includes both a data fidelity term and a smoothing term. The continuous problem is then discretized, and topographic effects are incorporated into the wind velocity field.

The article “Multi-Head Attention Refiner for Multi-View 3D Reconstruction”, by Kyunghee Lee, Ihjoon Cho, Boseung Yang, and Unsang Park, introduces a post-processing method called the Multi-Head Attention Refiner (MA-R), designed to improve the handling of object edges during the reconstruction process. The method integrates a multi-head attention mechanism into a U-Net-style refiner module. The proposed approach significantly enhances the reconstruction performance of Pix2Vox++ when multiple input images are used.

The article “Three-Dimensional Reconstruction of Indoor Scenes Based on Implicit Neural Representation”, by Zhaoji Lin, Yutao Huang, and Li Yao, addresses the problem of indoor scene reconstruction. The authors propose a 3D reconstruction method that combines Neural Radiance Fields (NeRFs) and Signed Distance Function (SDF) implicit representations. The volume density of the NeRF is leveraged to provide geometric information for the SDF field, while the learning of geometric shapes and surfaces is further enhanced through an adaptive normal prior optimization process.

The article “Single-Image-Based 3D Reconstruction of Endoscopic Images”, by Bilal Ahmad, Pål Anders Floor, Ivar Farup, and Casper Find Andersen, addresses 3D reconstruction from wireless capsule endoscopes (WCEs) designed for the examination of the human gastrointestinal (GI) tract. The authors propose a single-image reconstruction method using an artificial colon captured with an endoscope that mimics the behavior of a WCE. A Shape-from-Shading (SFS) algorithm reconstructs the 3D shape after geometric and radiometric calibration.

The article “Neural Radiance Field-Inspired Depth Map Refinement for Accurate Multi-View Stereo”, by Shintaro Ito, Kanta Miura, Koichi Ito, and Takafumi Aoki, proposes a method to refine depth maps obtained by Multi-View Stereo (MVS) through iterative optimization of Neural Radiance Fields (NeRFs). The proposed approach combines MVS and NeRFs to leverage the strengths of both in depth map estimation and employs NeRFs for depth map refinement. To further improve accuracy, the authors introduce a Huber loss into the NeRF optimization, which reduces estimation errors in the radiance fields by constraining errors larger than a threshold. The method is evaluated against conventional approaches, including COLMAP, NeRF, and DS-NeRF.

The article “Fast Data Generation for Training Deep-Learning 3D Reconstruction Approaches for Camera Arrays”, by Théo Barrios, Stéphanie Prévost, and Céline Loscos, focuses on 3D reconstruction from images captured by multi-camera arrays. The authors present a fully virtual data generator for creating large training datasets that can be adapted to any camera array configuration. The generator builds virtual scenes by randomly selecting objects and textures while following user-defined parameters such as disparity range or image properties (e.g., resolution, color space). Its effectiveness is validated by testing the generated datasets with established deep learning methods and depth reconstruction algorithms.
